# The role of PSA density in the MRI pathway for prostate cancer diagnostics

**DOI:** 10.1038/s41391-022-00579-6

**Published:** 2022-07-26

**Authors:** Hannes Cash, Martin Schostak

**Affiliations:** 1 Department of Urology, Otto-von-Guericke-University Magdeburg, Magdeburg, Germany; 2 PROURO, Berlin, Germany

**Keywords:** Outcomes research, Cancer screening

After initial resistance, the urological community has embraced the multiparametric MRI (mpMRI) within the diagnostic pathway of prostate cancer (PCa) [1]. Overall, this had led to a reduced number of men undergoing prostate biopsy and the detection rates for the men who do undergo targeted biopsy after a positive mpMRI (≥PI-RADS 3) are higher compared to systematic (Precision trial) [2]. Looking beyond the improvements of the MRI pathway, the detection rate for clinically significant PCa (csPCa), defined as ISUP Grade Group (GG) 2, is around 38–40% overall [2–4]. This seems rather low and is mostly driven by the low detection rate for PI-RADS 3 lesion (12% in the PRECISION trial compared to 60 and 83% for PI-RADS4/5) [2]. Therefore there is evident room for further improvement.

In this issue of Prostate Cancer and Prostatic disease Friesbie et al. show that PSA density (PSAd) is complementary to PI-RADS in detecting csPCa [5]. The study retrospectively included 327 men with 709 lesions who underwent both targeted and systematic biopsy. For the analysis, only the targeted biopsies (2 cores per lesion/mean of 4.3 cores per patient) were included to evaluate PSAd in a targeted only setting. The overall detection rate for csPCa in the cohort was 25 and 42% for all PCa. On a per patient analysis, a PSAd cuttof of ≥0.1 ng/ml/cc improved csPCa detection by 7% for PI-RADS 3, 17% for PI-RADS 4 and 15% for PI-RADS 5. The author did not present or discuss the benefits and risk of omitting biopsies and missed cancers according to their suggested risk stratification. The IMRIE study which retrospectively analyzed 2642 men showed that adding the standard PSAd (≥0.15 ng/ml/cc) to the MRI-pathway increased sensitivity and negative predictive value (NPV) for GG ≥ 2 (87.3–96.6% and 87.5–90.6%) [6]. Using a PSA density 0.12 ng/ml/ml improved sensitivity and NPV even further [6].

The novelty of the study of Friesbie et al. is that their analysis is based on a targeted-only approach, a concept gaining supporters. Although, currently the PI-RADS steering committee suggests a target saturation rather than a 2 core per lesion approach when performing targeted only biopsy. Further the authors explored an improved PSAd cutoff of ≥0.1 ng/ml/cc which lead to similar biopsy results for PI-RADS 3 with PSAd ≥0.1 ng/ml/cc compared to PI-RADS 4. The optimal PSAd threshold should therefore be further investigated to challenge the current PSAd of ≥0.15 ng/ml/cc in men undergoing targeted biopsy. First studies already evaluated the impact of different biopsy strategies according to PI-RADS/Likert and different PSAd cut-offs on omitted biopsies, missed GG 1 PCa and csPCA (i.e., I-RADS 4-5 or PI-RADS 3 if PSAd >0.10 or PSAd >0.2), 27% omitted biopsies, 24.4% missed GGG 1 and 4% missed csPCa) [7].

The clinical implications of this study by Friesbie et al. are that PSAd should be included when counseling men prior to targeted biopsy and may also be valid in a targeted-only setting.

Depending on the biopsy strategy used (targeted only vs. combined), a PSAd cutoff ≥0.15 ng/ml/cc can already reduce the number of men with PI-RADS 3 lesions to undergo biopsy [8]. Why is PI-RADS 3 so critical? First, the detection rate remains low and many men still undergo unnecessary biopsies. Second, depending on the radiologist’s experience the number of lesions rated PI-RADS 3 may vary depending on reader experience, even in the Academic setting which are the basis of most published data. This phenomenon probably is even greater in the community setting. In the PRECISION trial, the central MRI re-reading reduced the rate of PI-RADS 3 reading from 21 to 5% with a shift towards PI-RADS 1/2 compared to all sites [2]. Until artificial intelligence may standardize mpMRI reading even further, adding a simple and readily available clinical measure such as PSA density has the potential to reduce the number on men undergoing biopsy while increasing the rate of csPCa [9]. Initial studies also show benefits for adding PSAd into the decision process for repeat biopsy during Active Surveillance or during follow-up for men with a negative targeted biopsy [10, 11]. Variability of prostate volume measurement (MRI vs. TRUS) should be considered. Overall, the PSAd is ready at hand and does not involve further tests, we feel that PSAd is under-utilized in daily practice. The same under-utilization also applies to the available risk calculators, which already include PSA and prostate volume. The ERSPC-MRI calculator was recently tested in the study cohort of the PRECISION trial and showed that 13% of MRI scan would have been avoided, missing 7% csPCa and targeted biopsies reduced further by 9% missing one csPCa in the MRI arm [12]. Although the MRI pathway is leading toward precision medicine and risk stratification tools make personalized decision-making possible, the rate of missed csPCa that is acceptable remains debatable. Currently, we are caught in the dichotomy of either reducing the number men undergoing imaging/biopsies further and optimizing the rate of detected csPCa.
