# Correction to: The role of PSA density in the MRI pathway for prostate cancer diagnostics

**DOI:** 10.1038/s41391-023-00664-4

**Published:** 2023-04-03

**Authors:** Hannes Cash, Martin Schostak

**Affiliations:** 1 Department of Urology, Otto-von-Guericke-University Magdeburg, Magdeburg, Germany; 2 PROURO, Berlin, Germany

**Keywords:** Outcomes research, Cancer screening

Correction to: *Prostate Cancer and Prostatic Diseases* 10.1038/s41391-022-00579-6, 26 July 2022

The article “The role of PSA density in the MRI pathway for prostate cancer diagnostics”, written by Hannes Cash and Martin Schostak, was originally published electronically on the publisher’s internet portal on 26 July 2022 without open access. With the author(s)’ decision to opt for Open Choice the copyright of the article changed on 28 February 2023 to ©The Authors 2023 and the article is forthwith distributed under a Creative Commons Attribution 4.0 International License, which permits use, sharing, adaptation, distribution and reproduction in any medium or format, as long as you give appropriate credit to the original author(s) and the source, provide a link to the Creative Commons licence, and indicate if changes were made. The images or other third party material in this article are included in the article’s Creative Commons licence, unless indicated otherwise in a credit line to the material. If material is not included in the article’s Creative Commons licence and your intended use is not permitted by statutory regulation or exceeds the permitted use, you will need to obtain permission directly from the copyright holder. To view a copy of this licence, visit http://creativecommons.org/licenses/by/4.0.

